# Intermittent hypoxia treatment alleviates memory impairment in the 6-month-old APPswe/PS1dE9 mice and reduces amyloid beta accumulation and inflammation in the brain

**DOI:** 10.1186/s13195-021-00935-z

**Published:** 2021-11-29

**Authors:** Xiangpei Yue, Yanzhao Zhou, Meng Qiao, Xingnan Zhao, Xin Huang, Tong Zhao, Xiang Cheng, Ming Fan, Yongqi Zhao, Ruoli Chen, Lingling Zhu

**Affiliations:** 1 Beijing Institute of Basic Medical Sciences, Beijing, 100850 China; 2 Institute for Science and Technology in Medicine, School of Pharmacy, Keele University, Kelle, UK; 3 Co-innovation Center of Neuroregeneration, Nantong University, Nantong, 226019 China; 4 Hengyang Medical School, University of South China, Hengyang, 421001 Hunan China; 5 Anhui Medical University, Hefei, 230022 Anhui China

**Keywords:** Alzheimer’s disease, APPswe/PS1dE9 mice, Intermittent hypoxia treatment, Amyloid beta, BACE1, Neuroinflammation, Gene expression

## Abstract

**Background:**

Alzheimer’s disease (AD) is a progressive, degenerative, and terminal disease without cure. There is an urgent need for a new strategy to treat AD. The aim of this study was to investigate the effects of intermittent hypoxic treatment (IHT) on cognitive functions in a mouse model of AD and unravel the mechanism of action of IHT.

**Methods:**

Six-month-old APPswe/PS1dE9 (APP/PS1) male mice were exposed to hypoxic environment (14.3% O_2_) 4 h/day for 14 days or 28 days. Cognitive functions were measured by Morris water maze test after either 14 days or 42 days of interval. Thereafter the distribution of amyloid plaque and microglial activation were determined by mouse brain immunohistochemistry, while the amyloid beta (Aβ) and inflammatory cytokines were measured by ELISA and Western Blot. Microarray was used for studying gene expressions in the hippocampus.

**Results:**

IHT for 14 days or 28 days significantly improved the spatial memory ability of the 6-month-old APP/PS1 mice. The memory improvement by 14 days IHT lasted to 14 days, but not to 42 days. The level of Aβ plaques and neurofilament accumulations was reduced markedly after the IHT exposure. IHT reduced the pro-inflammatory cytokines IL-1β, IL-6 levels, and β-secretase cleavage of APP processing which implies reduced Aβ production. Microarray analysis revealed a large number of genes in the hippocampus were significantly altered which are known to be metabolism-regulated genes.

**Conclusions:**

This study provides evidence of the beneficial effect of IHT on the progression of AD by alleviating memory impairment, reducing Aβ accumulation and inflammation in the brain. IHT can be developed as a novel measure to relieve the progression of AD by targeting multiple pathways in the AD pathogenesis.

**Supplementary Information:**

The online version contains supplementary material available at 10.1186/s13195-021-00935-z.

## Background

Intermittent hypoxia is broadly defined as repeated episodes of hypoxia interspersed with episodes of normoxia [1]. Since the 1940s, intermittent hypoxia treatment (IHT) has been commonly used for training pilots, mountaineers, and athletes to improve performance by way of adaptation to reduce the oxygen environment [2–4]. In recent years, more and more research evidenced that IHT can be beneficial for the treatment of a wide range of diseases such as chronic heart and lung diseases, hypertension [5], iron-deficiency anemia [6], Parkinson’s disease, and ischemic coronary artery diseases [7–9]. As such, IHT has been applied for the treatment and prevention of chronic bronchial diseases (e.g., asthma) as a non-invasive, auxiliary treatment, via initiating a cascade of beneficial adaptive responses without adverse effects [10–14]. The cascade of IHT includes multiple changes in metabolic pathways and inflammatory responses and promotes angiogenesis and neurogenesis, which are evident at various levels, from systemic down to cellular [15–19]. However, the consequences of IHT on the pathological processes of Alzheimer’s disease (AD) are not defined.

AD is characterized by a progressive impairment of memory and other cognitive function [20, 21]. The incidence of AD is 15% among those 65 years and older and is close to 50% for those aged over 85 years [22]. The pathological hallmarks are extracellular amyloid beta (Aβ) accumulation and intracellular neurofibrillary tangles (NFTs), which leads to synaptic degeneration, neuroinflammation, eventually neuronal death [23–28]. Despite the significant progress that has been made toward understanding the genetic and molecular biological basis of AD in recent years, psychosocial and family nursing interventions, combined with acetylcholinesterase inhibitors and neuroprotective agents, are mainly used to alleviate AD symptoms, and there is no cure for AD yet [29]. It is critical to discover novel approaches, which could target multiple key pathways in the pathogenesis of AD, to halt the progression of AD.

In this context, we first validated and confirmed the in vivo effects of IHT on cognitive functions in APPswe/PS1-dE9 (APP/PS1) mice. APP/PS1 are double transgenic mice expressing a chimeric mouse/human amyloid precursor protein (Mo/HuAPP695swe) and a mutant human presenilin 1 (PS1-dE9), both directed to central nervous system neurons [30]. Both mutations are associated with early-onset AD [31]. We found that IHT alleviated the cognitive deficits, and inhibited Aβ production thus reducing Aβ fibrils in 6-month-old APP/PS1 mice, suggesting that IHT is an effective approach for inhibiting the onset of Aβ deposition. Furthermore, we identified the expression of a large number of genes in the mouse brain of the IHT group were altered, most of which were known to be metabolism-regulated genes or be enriched in p53 pathway.

## Methods

### Animals

Male, 8-week-old APP/PS1 mice and C57/BL6J wild-type mice were purchased from Beijing Huafukang Bioscience Co. Ltd. (Beijing, China) and were housed at a constant temperature under a 12-h light-dark cycle with unlimited access to standard diet and water in the animal house of the Beijing Institute of Basic Medical Sciences. All protocols involving the use of animals were conducted in accordance with the Institutional Animal Care and Use Committee of the Institute of Basic Medical Science. The mice were randomly assigned and all experiments were carried out double-blind.

### IHT exposure

The 6-month-old mice were placed in a decompression chamber (model: DYC-DWI; Fenglei, China) and were exposed to a hypoxic environment (14.3% O_2_, equal to 3000-m high altitude, at the velocity of 5 m/s in 10 min) 4 h per day for 14 days or 28 days (*n* = 10–12 per group). The control groups were placed in the chamber under normoxic conditions at the same time as the treatment groups and by the same experimenter. Routine blood test which measures the cells in the body through the blood was examined by an automated blood analyzer (SYSMEX, Kobe, Japan). Physiological indexes such as heart rate, breath rate, diastolic blood pressure (DBP), systolic blood pressure (SBP), mean blood pressure (MBP), and partial pressure of oxygen (PO_2_) were measured by a noninvasive pulse oximeter for animals (MouseOx® Plus pulse oximeter, PA, USA).

### Morris water maze test

The test was performed using a black circular pool of 122 cm in diameter that was divided into 4 equal quadrants. A transparent circular platform (10 cm^2^) was submerged 1.5 cm beneath the water surface. The water temperature was adjusted to 19–22 °C. The water maze activity was recorded using a video camera and analyzed with a professional analysis system (ANY-maze system, Dublin, Ireland). The experimental procedure includes the platform trial and the probe trial. The platform trial was performed for five consecutive days with a platform beneath the water, to measure the mice’s ability of learning and memory. Thereafter, the platform was removed from the pool, and the probe trial was performed to measure the memory retention of the spatial position of the platform (*n* = 10–12 per group).

### Immunohistochemistry

After the behavior test, the mice were deeply anesthetized with sodium pentobarbital (50 mg/kg, i.p.) and then were perfused transcardially with 0.9% sodium solution, followed by 4% paraformaldehyde (Solarbio, Beijing, China). Their brains were immediately removed, then rear-fixed while dehydrating with 30% sucrose-formaldehyde solution at 4 °C until they sank to the bottoms of 5 ml centrifuge tubes. The brains were embedded by optimal cutting temperature compound (O.C.T. compound) (SAKURA, CA, USA) and then were cut into 40-μm-thick coronal sections (*n* = 6 per group).

Polink-2 puls® Polymer HRP detection system (PV9001/PV9002, ZSGB-BIO, Beijing, China) was used to determine the distribution of amyloid plaques, neurofilament accumulations, and ionized calcium-binding adaptor molecule 1 (IBA1) immunoreactivity in mice brains. Briefly, the sections were pre-treated using heat mediated antigen retrieval with sodium citrate buffer (pH6, epitope retrieval solution 1) for 20 mins, then the sections were incubated in 3% hydrogen peroxide deionized water for 10 min to block endogenous peroxidase. After that, the sections were incubated respectively with primary antibody: mouse anti-amyloid precursor protein (APP)/Aβ (1:100, Cell Signaling Technology; USA), mouse anti-Neurofilament-L (1:100, Cell Signaling Technology; USA), and rabbit anti-IBA1 (1:200, WAKO, Japan), 4 °C overnight. Horseradish enzyme-labeled anti-mouse IgG (or anti-rabbit IgG) polymers were added to detect the primary antibodies and were visualized using an HRP detection system. Diaminobenzidine (DAB) (ZSGB-BIO, ZlI-9017, Beijing, China) was used as the chromogen. The section was then rinsed with tap water, re-dyed, dehydrated, transparent, and sealed. Images have been captured by a pathological section scanner (NDP, Japan). The number of Aβ-positive plaques in the cortex and hippocampus formation was calculated using Image Pro Plus 6.0 software. Briefly, 20 consecutive sections of each mouse cortex and hippocampus were imaged together and the areas and the total counts of Aβ-positive plaques in sections per six mouse brains of each group were determined using the software. The level of neurofilament accumulations and IBA1 immunoreactivity was measured by using the mean optical density of DAB staining.

The histofluorescence assay to detect the glycocalyx microvasculature was made with 10 μg/ml of Fluorescein Lycopersicon esculentum (Lectin) (Vectorlabs, Burlingame, USA). Brain slices were first washed three times with PBS and then incubated overnight with lectin (10 μg/ml) at 4 °C. The slices were washed three times with PBS and confocal microscope (Nikon, Tokyo, Japan) studies.

### Mouse brain homogenate

In separate mouse groups, after sacrifice, the mouse brain was taken out from the skull and mixed with protein extraction buffer which was made up of Protein lysate (Applygen, Beijing, China) and a protease inhibitor cocktail (EASYpack, Roche, Basel, Switzerland). The brain was then homogenized by high-speed tissue grinder (Servicebio, Beijing, China), and centrifuged with 12,000*g* at 4 °C for 10 min. The resulting supernatant was transferred to a new microcentrifuge tube and the protein concentrations were determined using a BCA protein assay kit following the manufacturer’s instructions.

### Enzyme-linked immunosorbent assay (ELISA)

The concentrations of Aβ_1-40_ and Aβ_1-42_ were determined by ELISA kits of Aβ_1-40_ (KHB344) and Aβ_1-42_ (KHB3481) (Invitrogen, California, USA). Tissues were treated with TBS and 5M guanidine/50 mM Tris HCL (pH 8.0) buffer before Aβ_1-40_ and Aβ_1-42_ ELISA measurement [32]. Changes in inflammatory cytokines (IL-1β, IL-6, TNFα) in the brain were detected by ELISA kits of IL-1β (SEA563MU), IL-6 (SEA079MU), TNFα (SEA133MU) (Cloud-Clone, TX, USA). All the ELISA kits were used in accordance with the manufacturer’s instructions (*n* = 6 per group).

### Western blotting

The hippocampus was dissected from mice brains and total protein was extracted. Equal amount of protein (20 μg) was denatured for 10 min in loading buffer at 95 °C, separated by 10–15% sodium dodecyl sulfate polyacrylamide gel electrophoresis (SDS-PAGE), and transferred to PVDF membrane (Bio-Rad, CA, USA) for 2 h at 4 °C. Membranes were blocked in 5% skim milk powder in TBS-T (TBS plus 0.1% Tween-20) for 1 h at room temperature. The membrane was then incubated with primary antibodies -- APP (1:1000), the components of the γ-secretase complex presenilin1 (PS1) (1:1000), beta-site APP-cleaving enzyme 1 (BACE1) (1:1000), Neurofilament-L (1:1000), Aβ (1:1000) (Cell Signaling Technology, MA, USA), a disintegrin and metalloprotease 10 (ADAM10) (1:1000) (Abcam, Cambridgeshire, UK), low-density lipoprotein receptor-related protein 1 (LRP1) (1:1000) (Cell Signaling Technology, MA, USA), Tau (1:1000) (Cell Signaling Technology, MA, USA), Tau-pS396 (1:1000) (Cell Signaling Technology, MA, USA), β-actin (1:10000) (Sigma-Aldrich, MO, USA) overnight at 4 °C. Followed by incubation with HRP-conjugated goat-anti-mouse or goat-anti-rabbit antibodies (1:10,000, Bio-Rad, CA, USA) for 1 h at room temperature, the membrane was detected using the ECL reagent (Bio-Rad, CA, USA). The band density was analyzed by Image J software (*n* = 6 per group).

### Microarray

A mouse genome 70-mer oligonucleotide microarray (*n* = 3 per group) was obtained from CapitalBio Corporation (Beijing, China) [33, 34]. RNA extraction, amplification, labeling, and hybridization microarray imaging and data analysis were performed according to the manufacturer’s instructions (CapitalBio Corp., Beijing, China). Statistical method *t* test Benjamini-Hochberg false discovery rate [BH-FDR] < 0.05, 2-fold change were used to screen out the significantly up- and downregulated genes after IHT. Gene Ontology (GO) Term annotation and Kyoto Encyclopedia of Genes and Genomes (KEGG) signaling pathway were used to analyze the function of altered genes.

### Quantitative real-time PCR

Total RNA was isolated from the three samples used in the microarray analysis, using TRIzol reagent according to the manufacturer’s instructions (Invitrogen, Carlsbad, CA, USA). Reverse transcription was performed by a reverse transcription kit (Vazyme Biotech Company Limited, China). cDNA was amplified by real-time PCR using SYBR Green master mix (Vazyme Biotech Company Limited, China) as recommended by the manufacturer. Gene expression was calculated relative to β-actin. Oligonucleotide primer sequences were listed in Table 1.

**Table 1:** QPCR primer sequences

Genes	Forward	Reverse
APP	5′-CCTTCTCGTTCCTGACAAGTGC-3′	5′-GGCAGCAACATGCCGTAGTCAT-3′
Bace1	5′-TGCTGCCATCACTGAATCGGAC-3′	5′-GGAATGTGGGTCTGCTTCACCA-3′
PS1	5′-GCAGTATCCTCGCTGGTGAAGA-3′	5′-CAGGCTATGGTTGTGTTCCAGTC-3′
Cdk5	5′-GTACTCCACGTCCATCGACATG-3′	5′-GCCATTGTTCCTCAGTCGGTGT-3′
Cdk5r1	5′-TCATCTCGGTGCTGCCTTGGAA-3′	5′-TTGGCACAGGACAGCGACTTCT-3′
Csnk1α1	5′-GATGTCCACTCCTGTTGAGGTG-3′	5′-AAGGATGCGGAATAGCTGCCTC-3′
Csnk1δ	5′-CCATCAACACGCACCTTGGCAT-3′	5′-CATACTTCTGCCTCTTGGTGGC-3′
Capn1	5′-CCTTGTTCAGCAAGTTGGCAGG-3′	5′-TCCAGGCTGAAGCCATTAGTGC-3′
β-actin	5′-ACTGTCGAGTCGCGTCCA-3′	5′-GTCATCCATGGCGAACTGGT-3′

*APP* amyloid precursor protein, *Bace1* beta-site APP-cleaving enzyme 1, *PS1* presenilin1, *Cdk5* cyclin-dependent kinase 5, *Cdk5r1* cyclin-dependent kinase 5 regulatory subunit 1, *Csnk1α1* casein kinase 1 alpha1, *Csnk1δ* casein kinase 1 delta, *Capn1* calpain1

### Data analysis

All data are presented as means ± the standard error of the mean (SEM). Unpaired *T* test was used to compare data between the control and IHT groups respectively. The level of statistical significance was set at *p* < 0.05. All graphs were generated using GraphPad 8.0 software.

## Results

### IHT alleviates cognitive impairment in 6-month-old APP/PS1 mice

#### IHT for 14 days or 28 days

**Fig. 1:**
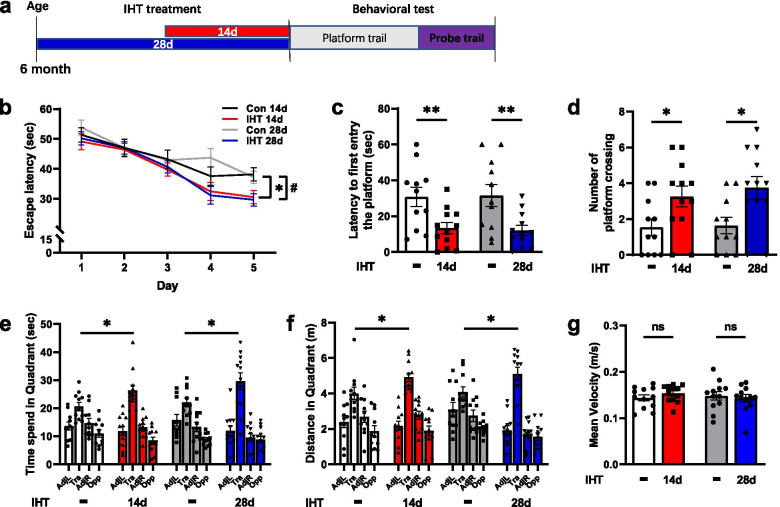
IHT for 14 days or 28 days alleviates memory impairment in APP/PS1 mice. **a** Schematic representation of the experimental design. 6-month-old APP/PS1 mice were exposed to a hypoxic environment (14.3% O_2_, 4 h/day) for 14 days or 28 days and then behavioral tests were performed. Mice with IHT had a shorter latency to find the platform (**b**), spent a significantly shorter time in first entry to the platform (**c**), as well as had significantly more crossing platform times in the probe trial at different time points (**d)**, longer time (**e**) and longer distance (**f**) in goal quadrant compared to the control mice. The IHT did not significantly change the mean velocity (**g**). Tra, Opp, AdjL and AdjR are the number of platform crossings in the training, opposite, adjacent left, and adjacent right quadrants, respectively. Control mice (*n* = 11), mice with IHT 14 days (*n* = 11), mice with IHT 28 days (*n* = 10). *p<0.05; **p < 0.01; ns, not significant

Six-month-old APP/PS1 transgenic male mice were subjected to intermittent hypoxia (14.4% O_2_, altitudes of 3000 m, 4 h/day) for 14 days or 28 days and then behavioral tests were performed (Fig. 1a). Wild type 6-month-old male C57/BL6J mice treated with the same IHT regime as the APP/PS1 showed no significant difference in the escape latency (*p* = 0.5243), the time to the first entry to the platform (*p* = 0.6746), the number of cross over the platform location (*p* = 0.9043), time spent (*p* = 0.6831), the distance traveled (*p* = 0.2319), and the mean velocity (*p* = 0.2684) in the goal quadrant compared to the control group mice (Additional file 1: Fig. S1, a to f). The results implied that IHT did not significantly affect the learning and spatial memory of the wild-type mice. IHT APP/PS1 mice showed a shorter latency to find the platform compared with APP/PS1 mice without IHT at day 5 (IHT 14 days: *p* = 0.0219; IHT 28 days: *p* = 0.0179) (Fig. 1b). APP/PS1 mice with IHT spent a significantly shorter time in first entry the platform compared with APP/PS1 mice without IHT (IHT 14 days: *p* = 0.0092; IHT 28 days: *p* = 0.0069) (Fig. 1c). APP/PS1 mice with IHT had significantly more crossing platform times in the probe trial at different time point compared with the mice without IHT (IHT 14 days: *p* = 0.0351; IHT 28 days: *p* = 0.0139) (Fig. 1d). Furthermore, APP/PS1 mice with IHT spent significantly longer time (IHT 14 days: *p* = 0.0332; IHT 28 days: *p* = 0.0396) and longer distance (IHT 14 days: *p* = 0.0429; IHT 28 days: *p* = 0.0489) in goal quadrant compared with normoxic mice (Fig. 1e, f). The results suggested that mice in the IHT group focused on looking for the platform in the goal quadrant.

The IHT did not significantly change the mean velocity (IHT 14 days: *p* = 0.2700; IHT 28 days: *p* = 0.6588) (Fig. 1g), the heart rate, breath rate, DBP, SBP, MBP, PO_2_, except the body weights which were significantly reduced by IHT 14 days and 28 days (Additional file 1: Table S1). Routine blood test was examined after IHT 14 days, and we found IHT increased hematocrit (HCT) (*p* = 0.0236) and hemoglobin (HGB) levels (*p* = 0.0116) (Additional file 1: Fig. S2, a to b), but did not significantly effect in the number of white blood cell (WBC) (*p* = 0.3911), red blood cell (RBC) (*p* = 0.4898), neutrophil (*p* = 0.5354), and platelet (*p* = 0.5053) (Additional file 1: Fig. S2, c to f). Both 14 days and 28 days of IHT significantly improved memory in 6-month-old APP/PS1 mice; however, there was no differences in behavior test (latency to first entry the platform: *p* = 0.7531, crossing platform times: *p* = 0.5646, time in goal quadrant: *p* = 0.3677, distance in goal quadrant: *p* = 0.6731, the mean velocity: *p* = 0.2852) between the two groups. Hence, the 14 days IHT was used only in the subsequent experiments.

#### IHT for 14 days with intervals of 14 days or 42 days

**Fig. 2:**
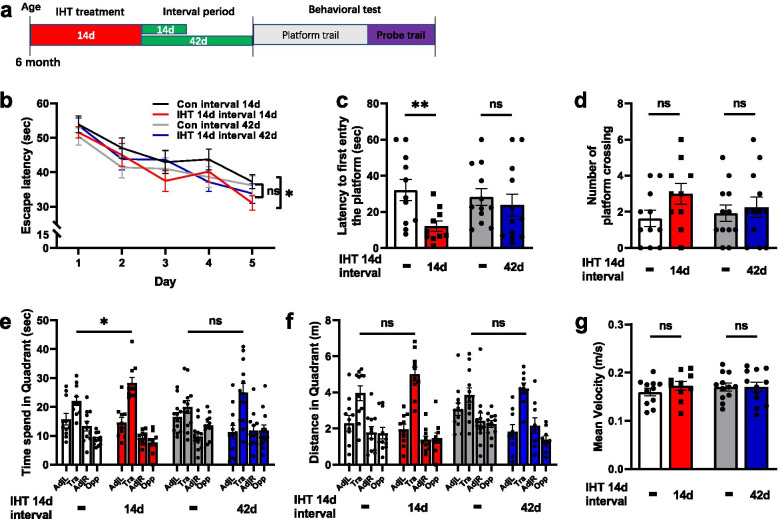
The effect of IHT for 14 days with intervals of 14 days or 42 days in APP/PS1 mice. **a** Schematic representation of the experimental design. Six-month-old APP/PS1 mice with 14 days IHT followed by different interval times (14 days or 42 days) and then behavioral tests were performed. There was a significantly shorter escape latency at day 5 (**b**), shorter time in the first entry of the platform (**c**), and longer time spent in the goal quadrant (**e**) after 14 days IHT following 14 days of interval but not 42 days of interval compared to the control mice. However, there was no significant difference in the number of crossing over the platform location (**d**), distance in goal quadrant (**f**), and mean velocity (**g**) in both intervals of 14-day and 42-day groups. Control mice (*n* = 11), mice with IHT 14 days following interval of 14 days (*n* = 10) in mice with IHT 14 days following interval 42 days (*n* = 10); *p<0.05; **p < 0.01; ns, not significant

To explore how long the memory improvement by IHT lasted, we next performed the behavior tests after 14 days IHT following different interval times (14 days or 42 days) in 6-month-old APP/PS1 mice (Fig. 2a). For the mice having 14 days IHT with 14 days of interval, the escape latency of the IHT group was significantly shorter compared to the control at day 5 (*p* = 0.0481) (Fig. 2b). Mice in IHT group spent a significantly shorter time in first entry the platform (*p* = 0.0086) (Fig. 2c), and longer time spent in the goal quadrant (*p* = 0.0187) (Fig. 2e). We observed no significant difference in the number of cross over the platform location (*p* = 0.0760) (Fig. 2d) and distance traveled (*p* = 0.0759) (Fig. 2f) between the control and 14 days IHT with 14-day interval mice. For the mice treated with 14 days IHT with 42 days of interval, we observed no significant difference in the escape latency (*p* = 0.0481) (Fig. 2b), in the time to first entry the platform (*p* = 0.5606) (Fig. 2c), number of cross over the platform location (*p* = 0.06497) (Fig 2d), and time spent (*p* = 0.2128) (Fig. 2e) and distance traveled (*p* = 0.3603) (Fig. 2f) in the goal quadrant compared to the control group mice. Similarly, IHT does not affect the mean velocity (interval of 14 days: *p* = 0.3054; interval of 42 days: *p* = 0.9411) (Fig.2g). This suggests that the memory improvement by 14 days IHT lasts to 14 days, but not to 42 days.

### IHT in 6-month-old APP/PS1 mice reduces amyloid load and inflammatory response

**Fig. 3:**
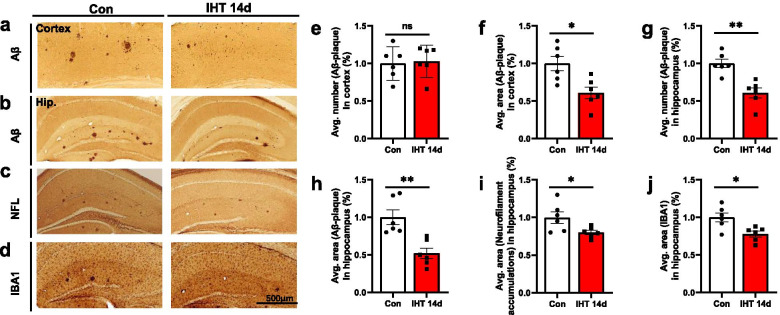
IHT reduces neuropathological changes in APP/PS1 mice. **a**–**d** Representative immunohistochemical images stained with antibodies against Aβ (**a**, **b**), Neurofilament-L (**c**), and IBA1(**d**). The Aβ plaque numbers were significantly reduced in the hippocampus (**g**), but not in the cortex (**e**), while the area of Aβ plaques was significantly decreased in both cortex (**f**) and hippocampus (**h**) in mice with IHT 14 days compared to the control ones. The area of neurofilament accumulation (**i**) and IBA1-positive cells aggregation (**j**) were significantly reduced in mice with IHT 14 days compared to the control ones. *n* = 6; *p<0.05; **p < 0.01; ns, not significant

Compared to the control mice, the APP/PS1 mice having 14 days IHT significantly decreased the number of Aβ immunoreactive plaques in the hippocampus (Fig. 3b), but not in the cortex (Fig. 3a). Quantification showed that APP/PS1 mice were subjected to IHT for 14 days significantly reduced plaque number by approximately 31% (*p* = 0.0012) in the hippocampus (Fig. 3g). While there was no significant change in plaque number (*p* = 0.8094) in the cortex (Fig. 3e). The beneficial effect of IHT on AD pathogenesis was further confirmed by counting the area of plaques in brain sections by image J analyses. The area of plaques in APP/PS1 mice were significantly decreased after 14 days IHT (cortex: *p* = 0.0104; hippocampus: *p* = 0.0026) (Fig. 3f, h).

We next examined the microglial reactivity in mouse brains using IBA1 and the neurofilament accumulations. IHT (14 days) APP/PS1 mice significantly decreased the accumulates area formed by neurofilament accumulations and IBA1 positive cells aggregation (Fig. 3c, d). Quantification showed that the accumulates area was reduced by nearly 20% (neurofilament accumulations: *p* = 0.0346; IBA1: *p* = 0.0122) in IHT group (Fig. 3i, j). There was no significant difference in lectin staining (p = 0.1993) (Additional file 1: Fig. S3). Lectins, which bind with high affinity to specific N-acetyl-D-glucosamine and poly-N-acetyl lactosamine sugar residues of endothelial plasma membrane glycocalyx, are commonly used markers for studying the endothelial cells [35]. In our study, there was no significant difference in vascular density in the APP/PS1 mouse brain between the IHT 14 days and the control groups, this suggested that IHT may have no significant effect on vascular structure.

**Fig. 4:**
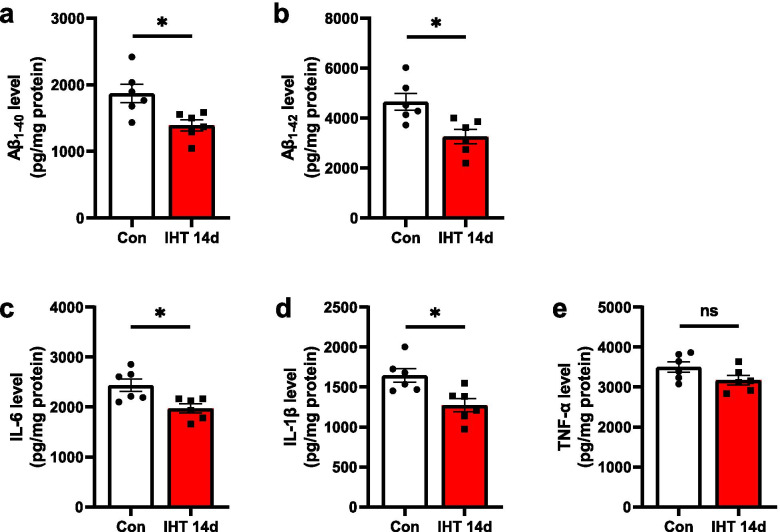
IHT decreases the levels of Aβ_1-40_ (Aβ_1-42_) and the pro-inflammatory cytokines in the brain of APP/PS1 mice. Mice with IHT 14 days had reduced levels of Aβ_1-40_ (**a**) and Aβ_1-42_ (**b**), IL-6 (**c**), and IL-1β (**d)** in the brain compared to the control mice. However, the level of TNF-α was not changed by the IHT (**e**). *n* = 6; *p<0.05; ns, not significant

Then, we performed ELISA to analyze the levels of Aβ_1-40_ and Aβ_1-42_ in the hippocampus in mice with IHT 14 days. The levels of Aβ_1-40_ and Aβ_1-42_ were reduced to 75.96 ± 4.4% and 70.08± 7.52% respectively compared to controls (Aβ_1-40_: *p* = 0.0135; Aβ_1-42_: *p* = 0.0107) (Fig. 4a, b). These data indicate that IHT reduces the formation of plaques and Aβ production in the AD mice. We then examined changes in the pro-inflammatory cytokines IL-1β, IL-6, and TNF-α after exposure to IHT for 14 days. The result indicated that IHT down-regulated the level of IL-6 (*p* = 0.0125) (Fig. 4c) and IL-1β (*p* = 0.0110) (Fig. 4d) but did not affect the level of TNF-α (*p* = 0.0919) (Fig. 4e) in the brain tissue. These results demonstrate that IHT reduces the inflammatory response. In our study, 14 days IHT reduces the plaque load and inflammatory in APP/PS1 mice, but we did not observe the effect on the plaque load and inflammatory with an interval of 14 days.

**Fig. 5:**
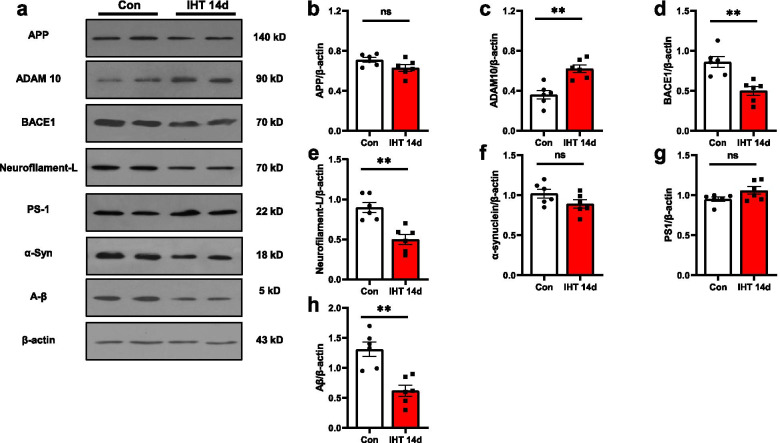
IHT affects the amyloidogenic proteins APP processing. **a** Representative Western blots of APP, ADAM10, BACE1, Neurofilament-L, PS1, α-Syn, and Aβ. There was a significant increase of ADAM10 (**c**) and decrease of BACE1 (**d**) and Neurofilament-L (**e**) in mouse brain with IHT 14 days compared to the control mice. The expression of APP (**b**), α-Synuclein (**f**) and PS1 (**g**) in brain tissue was not significantly changed by the IHT. *n* = 6; **p < 0.01; ns, not significant

We next analyzed molecules related to the degradation of APP via Western blotting. The expression of APP (*p* = 0.0775), PS1 (*p* = 0.0751), and α-Synuclein (*p* = 0.1166) (Fig. 5b, f, g) in brain tissue was not significant difference after IHT for 14 days, but we observed markedly increased ADAM10 (*p* = 0.0011) (Fig. 5c) and reduced BACE1(*p* = 0.0018) levels (Fig. 5d) in APP/PS1 mice with IHT 14 days, while the expression of Neurofilament-L (*p* = 0.0011) (Fig. 5e) and Aβ (*p* = 0.001) both down-regulated. We also measured Tau and LRP1 protein levels, and found Tau-pS396 was reduced after IHT (IHT 14 days: *p* = 0.0093, IHT 28 days: *p* = 0.0205) (Additional file 1: Fig. S4) but there was no statistical difference of LRP1 level between two groups (*p* = 0.1458) (Additional file 1: Fig. S5). Taken together, these results demonstrate that IHT inhibits amyloidogenic APP processing.

### Microarray analysis of gene expression in APP/PS1 mice after IHT

**Fig. 6:**
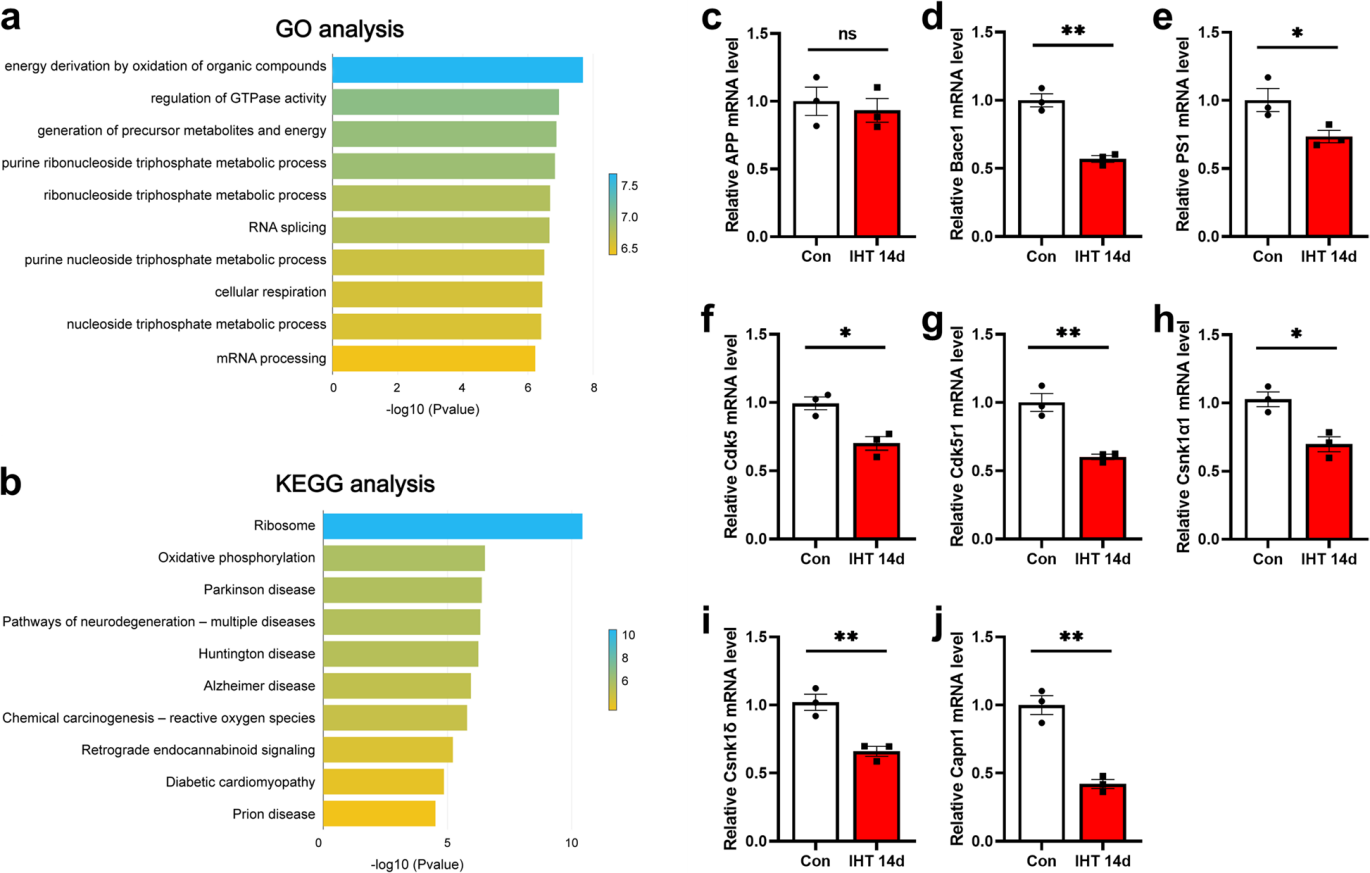
Microarray and qPCR analysis of differentially expressed genes in the hippocampus of APP/PS1 mice with IHT 14 days compared to the control ones. **a** Top enriched GO terms for the differentially expressed genes. **b** Top enriched KEGG pathways for the differentially expressed genes. The color represents the *P*-value. **c**–**j** were down-regulated genes by the IHT (**c**) APP, (**d**) Bace1, (**e**) PS1, (**f**) Cdk5, (**g**) Cdk5r1, (**h**) Csnk1α1, (**i**) Csnk1δ, and (**j**) Capn1. *n* = 3; *p <0.05; **p < 0.01; ns, not significant

To determine the gene expression profile in the hippocampus of APP/PS1 mouse after IHT, we performed microarray analysis representing 6,347 genes. Generally, there were 261 genes that were significantly up- and downregulated after IHT (*t* test BH FDR< 0.05, 2-fold change). GO term annotation and KEGG signaling pathway were used to analyze the function of altered genes. The results showed that the most significant genes were mainly associated with energy derivation by oxidation of organic compounds, regulation of GTPase activity, and some metabolic processes at the biological process level (Fig. 6a). Among the top, the changed number of genes in GO annotations after IHT were “metabolic process,” “cellular process,” and “biological regulation” (Additional file 1: Table S2). In addition to annotation the function of genes, genes are involved in various pathways. The most significant pathways of enriched genes were ribosome, oxidative phosphorylation, and some neurodegenerative diseases involved AD (Fig. 6b). Besides, the pathways with more enriched genes were Glycolysis/Gluconeogenesis and Citrate cycle (TCA cycle) (Additional file 1: Table S3). In general, the large numbers of genes with significantly altered in IHT APP/PS1 mice are known to be metabolism-regulated genes.

Glycogen synthase kinase 3 beta (Gsk-3β) and cyclin-dependent kinase 5 (Cdk5) are key molecules in the development of AD [36, 37]. GSK-3β, a major Tau kinase, has been found to be overactivated in the brains of AD patients [38, 39], it is responsible for the hyperphosphorylation of tau, which is an important component of NFTs, making it play a key role in the pathogenesis of AD [40]. GSK-3β is also involved in Aβ-induced toxicity through different mechanisms, it has been reported that Aβ blocks Wnt-mediated GSK-3β-inhibition leading to an increase in Aβ formation and tau hyperphosphorylation [41]. Further, GSK-3β is expressed in both microglia and astrocytes, as a modulator of inflammatory cytokine levels in the brain, promotes the production of cytokines [42]. A surprising result from the microarray study was that the expressions of several key genes involved in the regulation of AD pathology include the genes encoding microtubule-associated protein (Tau), the components of the γ-secretase complex PS1, Gsk-3β, protein phosphatase 2 (Ppp2ca), Cdk5, Cdk5r1, Capn1, Csnk1α1, and Csnk1δ decreased.

Quantitative PCR was performed to validate the altered expression levels of these genes using cDNA derived from total RNA of the brain tissue samples. There was a good agreement between the microarray and Q-PCR results (Fig. 6c–j). Relative quantitative analysis of gene expression was performed: APP (*p* = 0.6489) (Fig. 6c), Bace1 (*p* = 0.0013) (Fig. 6d), PS1 (*p* = 0.0490) (Fig. 6e), Cdk5 (*p* = 0.0129) (Fig. 6f), Cdk5r1 (*p* = 0.0043) (Fig. 6g), Csnk1α1 (*p* = 0.0125) (Fig. 6h), Csnk1δ (*p* = 0.0066) (Fig. 6i), and Capn1 (*p* = 0.0016) (Fig. 6j). Consistently, the protective role of IHT on APP/PS1 mice probably acts on APP processing and decreases Aβ production by inhibiting the GSK3β pathway.

Interestingly, most of the genes from our signature that are down-regulated after IHT such as Capn1, Csnk1δ, Csnk1α1, Cdk5, and Cdk5r1, belong to p53 related pathway. Increasing evidence has suggested p53 pathway plays a pivotal role in AD, implying that modulation of cell death pathways might be of therapeutic benefit in IHT and indeed in age-related neurological disorders.

## Discussion

**Fig. 7:**
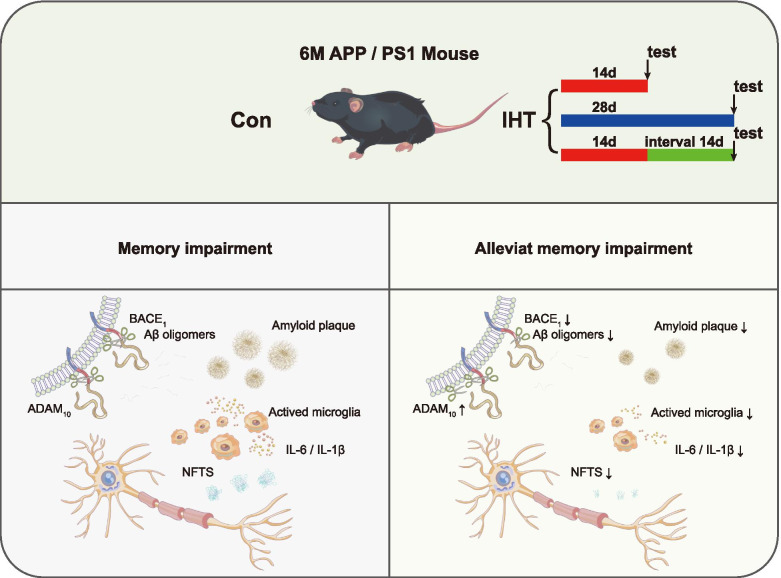
Working model of IHT alleviates memory impairment in 6-month-old APP/PS1 mice, reduces amyloid accumulation and inflammation in the brain. IHT for 14 days or 28 days, 14 days with interval of 14 days alleviates memory impairment in APP/PS1 mice. IHT reduced the pro-inflammatory cytokines IL-1β, IL-6 levels, and increased ADAM10 and decreased BACE1 of APP processing thus decreasing Aβ production

In this study, we provided evidence of the beneficial effect of IHT on the APP/PS1 mice as shown in the working model (Fig. 7). We demonstrated that 14 days IHT inhibited Aβ production, reduced neuritic plaque formation, and alleviated the memory deficits in 6-month-old APP/PS1 mice, suggesting that IHT could be used for the prevention/treatment of AD. We further showed IHT alleviated AD pathogenesis by reducing β-secretase cleavage of APP processing thus decreasing Aβ production in the brain tissue. In addition, we found a large number of genes expression were significantly altered by IHT, most of which were known to be metabolism-regulated genes or be enriched in p53 pathway, implying that modulation of metabolism and cell death by IHT might be of therapeutic benefit in AD.

AD is a progressive neurodegenerative disease characterized by cognitive decline and behavioral changes [43]. Formation of amyloid plaques and NFTs are two central hallmarks in the AD. Amyloid plaques are the dense composites of Aβ which accumulates around the nerve cells, while NFTs are the twisted fibers containing hyperphosphorylated tau proteins present in certain residues of Aβ that build up inside the brain cells [44, 45]. The epitope defined by phosphorylation of S396 in tau protein is strongly implicated in AD-associated tau pathology [46]. We found IHT could reduce Tau-pS396 protein levels and NFTs density in the APP/PS1 mice. High levels of fibrillary Aβ in the brain are associated with neuronal and synaptic loss and impairment of neuronal functions [47]. Aβ is a cleaved product of the APP via proteolysis by BACE1 and γ-secretase [48]. BACE1 is a prime drug target for slowing down Aβ production in early AD [49]. In AD, excessive accumulation of Aβ monomers results in their assembly into soluble, diffusible oligomers e.g. Aβ dimers which directly induce tau hyperphosphorylation and neurite degeneration [50]. In this study, we found IHT could reduce BACE1 while increasing α-secretase levels resulting in reduced Aβ plaque and deposition in 6-month-old APP/PS1 mice. This is consistent with a number of studies that demonstrate cognitive improvement following inhibition of Aβ generating enzymes in mouse models of AD [51, 52]. LRP1 is highly expressed by neurons and has multiple functions in the regulation of cerebrovascular homeostasis and AD’ pathology. Many evidences have indicated that LRP1 mediates brain Aβ clearance and amyloid deposition [53–56], but in the present study, IHT did not significantly affect LRP1 protein expression.

It has been demonstrated that there are increased amyloid brain levels in APP/PS1 mice since 3 months old while strong plaque deposition can be detected at 6 months [57–59]. The previous studies from our and other groups have identified that Aβ aggregation was correlated with memory deficits in the APP/PS1 mice [60–62]. Consistent with this finding, we found 14 days of IHT significantly reduced Aβ protein expression and Aβ aggregation, improved the cognitive performance in APP/PS1 mice. The results indicated that IHT may serve as a protective role of Aβ-induced AD pathological processes.

The protein neurofilament-L is a key component of axons and the main byproduct of nerve cell degeneration. Research studies have shown neurofilament accumulations in many human neurological disorders including Parkinson’s disease, AD, Charcot-Marie-Tooth disease, and Amyotrophic Lateral Sclerosis [63]. It has been found in both cerebrospinal fluid and blood in AD patients [64, 65]. Neurofilament-L has been used as a biomarker and may monitor efficacy of disease-altering drugs in clinical trials with AD patients [65–69]. In our study, the expression of neurofilament-L protein was reduced by nearly 45% after IHT 14 days. IHT was found to promote hippocampal neurogenesis [15], to prevent overproduction of nitric oxide in the brain and neurodegeneration induced by Aβ toxicity [70], and to enhance brain-derived neurotrophic factor (BDNF) expression in 9 months APP/PS1 mice [71].

Neuroinflammation, the inflammatory response of the central nervous system, can be caused as a result of Aβ plaques deposition and contributes to the progression of AD [72–74]. Although neuroinflammation has roles in protecting the brain, prolonged neuroinflammation has been known to increase Aβ burden and the plaque deposition creates a vicious cycle where the Aβ load is increased which further results in microglia activation and subsequent neuroinflammation [73]. Increased expression of inflammatory mediators in postmortem brains of people with AD and epidemiological studies link the use of anti-inflammatory drugs with reduced risk for the disorder [75–80]. Nonsteroidal anti-inflammatory drug treatment can reduce brain Aβ levels, amyloid plaque burden, and microglial activation in an animal model of AD [81]. CD11b-positive microglia and glial fibrillary acidic protein (GFAP) positive astrocytes were first detected at 3 months old APP/PS1 mice with a close association with amyloid plaques [82]. We found IHT (14 days) significantly decreased the area of plaques formed by IBA1 positive cells aggregation in APP/PS1 mice. IBA1 is specifically expressed in macrophages/microglia antibody and the area of plaques formed by IBA1 positive cells aggregation shows the microglial reactivity [83]. The brains of APP/PS1 mice were under an active inflammatory stress, the amplitude of cytokine response TNF-α, IL-6, and other inflammatory factors was dependent on Aβ levels [84]. IHT reduced the pro-inflammatory cytokines in mice: IL-1β (*p* < 0.05), IL-6 (*p* < 0.05), and TNF-α (*p* > 0.05) in their protein expressions in the brain. These data indicated that the effect of IHT on APP/PS1 mice might be related to an inflammatory response.

Oxygen is vital to maintain the normal functions of almost all the organs [85]. The important roles of oxygen are not only reflected in the development, but also in the pathological processes of many cerebral diseases [16]. Oxygen supply in brain tissue plays a key role in neurodegeneration during the aging process [86]. Previously studies reported that acclimatization to hypoxia when ascent to altitude is reflected by progressive increases in ventilation, adaptations in the cardiovascular system that enhance oxygen delivery to tissues for better extraction of oxygen, and more efficient utilization of oxygen for metabolic processes from the integrative systems to the molecular and genomic level [19]. These adaptive responses raise the potential therapeutic possibility of IHT on these neurodegenerative diseases. There is also epidemiological evidence that resistance to altitude may impact the risk of death rates in AD, which each doubling of altitude was associated with a 2 percent reduction in Alzheimer’s death rates [87]. A number of studies examined IHT’s safety and therapeutic efficacy in elderly patients with mild cognitive impairment (MCI), a precursor symptom of AD. They suggest that adaptation to moderate IHT may enhance cerebral oxygenation, improve cognitive function, and decrease circulating biomarkers of AD [88, 89].

The beneficial effects of IHT to promote memory deficits in the 6-month-old APP/PS1 mice are due in part to its ability to induce changes in gene transcription. We found that the expression of a wide variety of genes involved in IHT on AD pathogenesis is largely due to the regulation of Glycolysis/Gluconeogenesis, TCA cycle, APP processing, and cell death. The most significant pathways of enriched genes were ribosomes. Ribosomes are the cellular factories responsible for making proteins. It is the ribosome that reads the sequence of mRNA and translates it into the correct sequence of amino acids for each protein. Although no data are available regarding the precise mechanism of IHT on neurodegenerative diseases, it provides new directions for neurology aimed at understanding mechanisms of IHT on aged-related diseases. Other mechanisms that could account for the effective roles of IHT need further investigation.

There are several different experimental models to study IHT in animals. The different protocol also has different effects on various diseases mainly depending on the intervals and the altitude of hypoxic exposures, the number of hypoxic episodes per day, and the number of exposure days. Goryacheva et al. demonstrated IHT (4000 m for 14 days, 4 h per day) significantly alleviated the memory deficiency in rats with Aβ_25-35_ being injected into the basal macronuclei of the brain [70]. Suzuki discovered short-duration of IHT (4 cycles of 12–13% O_2_ for 15 min and 20.9% O_2_ for 10 min) for 28 days facilitated endurance exercise performance in mice [90].

On the contrary, Snyder et al. found increased oxidative stress and inflammation in a manner consistent with early stages of neurodegenerative disease in rats were exposed to 7 days chronic intermittent hypoxia for 8 h/day [91]. Shiota et al. reported that chronic intermittent hypoxia/reoxygenation (5% O_2_ and 21% O_2_ every 10 min, 8 h/day for 4 weeks) facilitates amyloid-β generation in AD model mice [92].

Recently, more and more studies have shown that some non-invasive stimuli can improve the cognitive behavior and pathological changes of AD mouse models. The methods were different but showed similar positive results. A non-invasive light flicker (gamma entrainment using sensory stimulus or GENUS) furthermore, combined auditory and visual GENUS, have been reported to improve spatial and recognition memory and reduce amyloid in AC and hippocampus of 5XFAD mice [93, 94]. Hyperbaric oxygen therapy (HBOT) attenuated neuroinflammatory processes and reduced amyloid burden, and tau phosphorylation in 3xTg mice, and ameliorated their behavioral deficits [32]. HBOT did the opposite of our study but showed very similar effects in terms of reducing beta amyloid pathology. The reason may be that IHT and HBOT both have wide-ranging effects on the brain to improve cognitive function through different mechanisms [95].

Previous studies of our lab showed that the proliferation of neural stem cells in the hippocampal subventricular zone (SVZ) and dentate gyrus (DG) was significantly increased in adult rats with IHT for 2 weeks at 3000 m altitude [96]. In vivo real-time oxygen probe detection revealed that oxygen content in the hippocampal DG region fluctuated significantly after IHT, and promote the growth of dendritic spines of hippocampal neurons [17]. IHT promotes neurogenesis leading to more newborn neurons in the hippocampus in adult rats and stably enhanced the expression of BDNF to relieve the depressant-like effects in animal models, suggesting that IHT is involved in BDNF signaling [15]. In contrast, chronic or severe hypoxia significantly alters neurotransmitter systems and produces progressive brain injury and subsequent impairment of neural function [97, 98]. This may be explained to the adverse effect of different episodic hypoxia to differentiate between physiological and pathological responses in neurodegenerative diseases.

## Limitations

This study has several limitations as follows: (i) it is well established that inflammation and hypoxia-induced pathways are different between sex, while we used only male AD mice in this study for reducing variants in the experiments. It remains to be studied whether IHT has similar effects on female APPswe/PS1dE9 mice; (ii). considering the timeliness of IHT, we only adopted the water maze to test the spatial learning and memory ability of mice. The results will be more solid if other behavioral tests can be used without affecting the timeliness of IHT, for example, the Y-maze test and novel object location etc.; (iii). hypoxia is a double-edged sword, severe and persistent hypoxia can cause damage to the body, while mild and temporary hypoxia can bring beneficial effects to the body. Our study represents a proof of concept; however, the duration of IHT can be further optimized; (iv). since AD is a chronic neurodegenerative disease, further studies are needed for validating these discoveries in the clinical setting.

## Conclusion

In conclusion, this study demonstrates that IHT (14 days and 28 days) improves spatial memory ability in 6-month-old APP/PS1 mice. The improvement in spatial memory ability lasts to 14 days but not 42 days of interval, indicating the beneficial effect of IHT is temporary. The spatial memory ability improvement by IHT is associated with reduced formation of amyloid plaques and neurofibrillary tangles, as well as inhibition of neuroinflammation, and changes in gene expression in the hippocampus. The genes that are down-regulated after IHT such as Capn1, Csnk1δ, Csnk1α1, Cdk5, and Cdk5r1, belong to p53 related pathway, suggesting the p53 pathway plays a pivotal role in the process. Our study has further clarified the therapeutic effect of IHT on APP/PS1 mice, and explored the multi-target regulation mechanism of its intervention effect. Although further research is needed to elucidate the underlying beneficial mechanisms of IHT, we suggest that IHT presents a new means for treating AD.

## Supplementary Information

**Additional file 1: Table S1.** The physiological parameters in IHT APP/PS1 mice. **Table S2.** List of GO terms that were significantly affected by IHT in APP/PS1 mice. **Table S3.** List of KEGG pathway that were significantly affected by IHT in APP/PS1 mice. **Figure S1.** Effects of IHT on the behavior of wild-type C57/BL6J mice. There was no significant difference in the escape latency (a), the time to first entry the platform (b), number of cross over the platform location (c), time spent (d), the distance traveled (e) and the mean velocity (f) in the goal quadrant in C57/BL6J mice with IHT 14d compared to the control group mice. *n* = 12 per group. ns, not significant. **Figure S2.** Effect of IHT on the blood test in APP/PS1 mice. There was no significant difference in (a) HCT: hematocrit, (b) HGB: hemoglobin, (c) WBC: white blood cell, (d) RBC: red blood cell, (e) Neutrophil, (f) Platelet in the blood of APP/PS1 mice with IHT 14d compared to the controls. *n* = 5 per group. ns, not significant. **Figure S3.** Representative immunohistochemical images stained with Lectin in APP/PS1 mouse brain sections. There was no significant difference in vascular density in the APP/PS1 mouse brain between the IHT 14d and the control groups. *n* = 6 per group. ns, not significant. **Figure S4.** Representative Western blots of Tau-pS396 and total Tau in mouse hippocampus homogenate. There was significant decrease of the ratios of Tau-pS396 / total Tau protein expression level in APP/PS1 mouse brain between IHT 14d and 28d compared to the control groups. *n* = 4 per group; *p <0.05; **p < 0.01. **Figure S5.** Representative Western blots of LRP1 in mouse hippocampus homogenate. There was no significant difference of the LRP1 protein expression level in APP/PS1 mouse brain between IHT 14d compared to the control groups. n = 6 per group. ns, not significant.

## Data Availability

The datasets used for the analyses are available from the corresponding author on reasonable request.

## Abbreviations

AD: Alzheimer’s disease
IHT: Intermittent hypoxic treatment
APP/PS1: APPswe/PS1-dE9
Aβ: Amyloid beta
NFTs: Neurofibrillary tangles
DBP: Diastolic blood pressure
SBP: Systolic blood pressure
MBP: Mean blood pressure
PO_2_: Partial pressure of oxygen
IBA1: Ionized calcium-binding adaptor molecule 1
DAB: Diaminobenzidine
O.C.T. compound: Optimal cutting temperature compound
APP: Amyloid precursor protein
SDS-PAGE: Sodium dodecyl sulfate polyacrylamide gel electrophoresis
PS1: Presenilin1
BACE1: Beta-site APP-cleaving enzyme 1
ADAM10: A disintegrin and metalloprotease 10
LRP1: Low-density lipoprotein receptor-related protein 1
Cdk5: Cyclin-dependent kinase 5
Cdk5r1: Cyclin-dependent kinase 5 regulatory subunit 1
Csnk1α1: Casein kinase 1 alpha1
Csnk1δ: Casein kinase 1 delta
Capn1: Calpain1
TCA cycle: Citrate cycle
Gsk-3β: Glycogen synthase kinase 3 beta
Ppp2ca: Protein phosphatase 2
BDNF: Brain-derived neurotrophic factor
GFAP: Glial fibrillary acidic protein
MCI: Mild cognitive impairment
HBOT: Hyperbaric oxygen therapy
SVZ: Subventricular zone
DG: Dentate gyrus
