# Supplementary material for: Intermittent hypoxia treatment alleviates memory impairment in the 6-month-old APPswe/PS1dE9 mice and reduces amyloid beta accumulation and inflammation in the brain
Source: Alzheimers Res Ther. 2021 Nov 29;13:194. doi: 10.1186/s13195-021-00935-z (PMC8630860; doi:10.1186/s13195-021-00935-z)
Supplement: Supplementary file 1 — Additional file 1: Table S1. The physiological parameters in IHT APP/PS1 mice. Table S2. List of GO terms that were significantly affected by IHT in APP/PS1 mice. Table S3. List of KEGG pathway that were significantly affected by IHT in APP/PS1 mice. Figure S1. Effects of IHT on the behavior of wild-type C57/BL6J mice. There was no significant difference in the escape latency (a), the time to first entry the platform (b), number of cross over the platform location (c), time spent (d), the distance traveled (e) and the mean velocity (f) in the goal quadrant in C57/BL6J mice with IHT 14d compared to the control group mice. n = 12 per group. ns, not significant. Figure S2. Effect of IHT on the blood test in APP/PS1 mice. There was no significant difference in (a) HCT: hematocrit, (b) HGB: hemoglobin, (c) WBC: white blood cell, (d) RBC: red blood cell, (e) Neutrophil, (f) Platelet in the blood of APP/PS1 mice with IHT 14d compared to the controls. n = 5 per group. ns, not significant. Figure S3. Representative immunohistochemical images stained with Lectin in APP/PS1 mouse brain sections. There was no significant difference in vascular density in the APP/PS1 mouse brain between the IHT 14d and the control groups. n = 6 per group. ns, not significant. Figure S4. Representative Western blots of Tau-pS396 and total Tau in mouse hippocampus homogenate. There was significant decrease of the ratios of Tau-pS396 / total Tau protein expression level in APP/PS1 mouse brain between IHT 14d and 28d compared to the control groups. n = 4 per group; *p <0.05; **p < 0.01. Figure S5. Representative Western blots of LRP1 in mouse hippocampus homogenate. There was no significant difference of the LRP1 protein expression level in APP/PS1 mouse brain between IHT 14d compared to the control groups. n = 6 per group. ns, not significant. [file 13195_2021_935_MOESM1_ESM.docx]

**Table S1. The physiological parameters in IHT APP/PS1 mice**

| Parameters | Normoxia | IHT-14d | IHT-28d |
| --- | --- | --- | --- |
| Heart rate (bpm)  Breath rate (bpm)  MBP (mmHg)  SBP (mmHg)  DBP (mmHg)  PO_2_ (mmHg)  Body weight (g) | 601.37±19.57  95.02±7.16  85.02±3.31  120.00±2.31  67.30±4.39  88.45±1.37  31.6±0.9 | 595.77±22.04  98.63±5.89  89.8±1.94  122.63±1.66  71.23±2.21  85.50±1.50  29.6±0.9* | 590.98±14.44  96.63±4.35  84.73±1.56  116.98±1.77  68.75±1.48  86.20±2.49  29.5±0.2* |

****p*<0.05 compare with normoxia group, n=8**

| **Table S2. List of GO terms that were significantly affected by IHT**  **in APP/PS1 mice** | |
| --- | --- |
| **Gene Ontology Term** | **Changed number of genes** |
| GO 0008152 metabolic process | 147 |
| GO 0009987 cellular process | 102 |
| GO 0065007 biological regulation | 97 |
| GO 0044237 cellular metabolic process | 92 |
| GO 0009058 biosynthetic process | 53 |
| GO 0006810 transport | 42 |
| GO 0051234 establishment of localization | 37 |
| GO 0006807 nitrogen compound metabolic process | 37 |
| GO 0032502 developmental process | 35 |
| GO 0050896 response to stimulus | 31 |

| **Table S3. List of KEGG pathway that were significantly affected by IHT in APP/PS1 mice** | |
| --- | --- |
| **Category** | **Number of enriched genes** |
| \| Glycolysis / Gluconeogenesis \| \| --- \| | 8 |
| Citrate cycle (TCA cycle) | 8 |
| Lysosome | 6 |
| Ubiquitin mediated proteolysis | 8 |
| \| Neurotrophin signaling pathway \| \| --- \| | 7 |
| Pyruvate metabolism | 6 |
| Endocytosis | 8 |
| Wnt signaling pathway | 6 |

**Figure. S1**


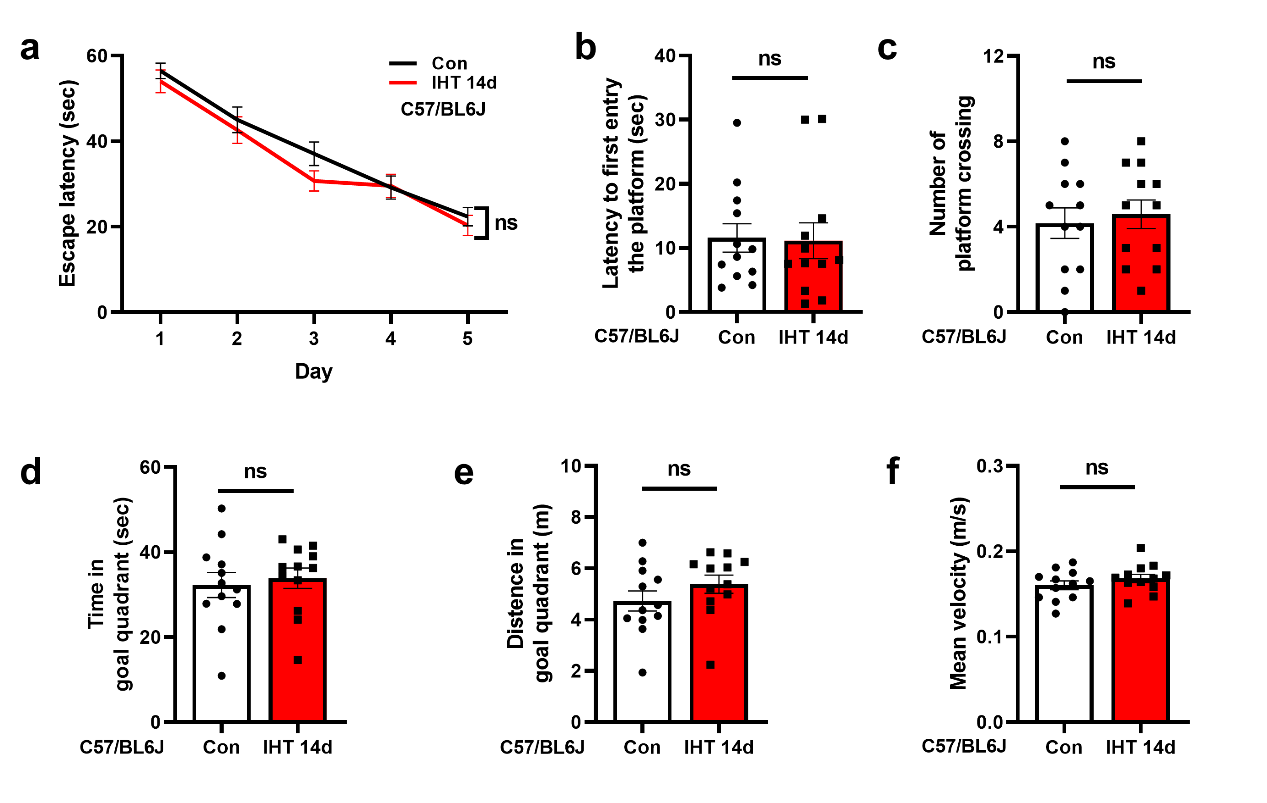


**Fig. S1** Effects of IHT on the behavior of wild-type C57/BL6J mice. There was no significant difference in the escape latency **(a)**, the time to first entry the platform **(b)**, number of cross over the platform location **(c)**, time spent **(d),** the distance traveled **(e)** and the mean velocity **(f)** in the goal quadrant in C57/BL6J mice with IHT 14d compared to the control group mice. n=12 per group. ns, not significant.

**Figure. S2**


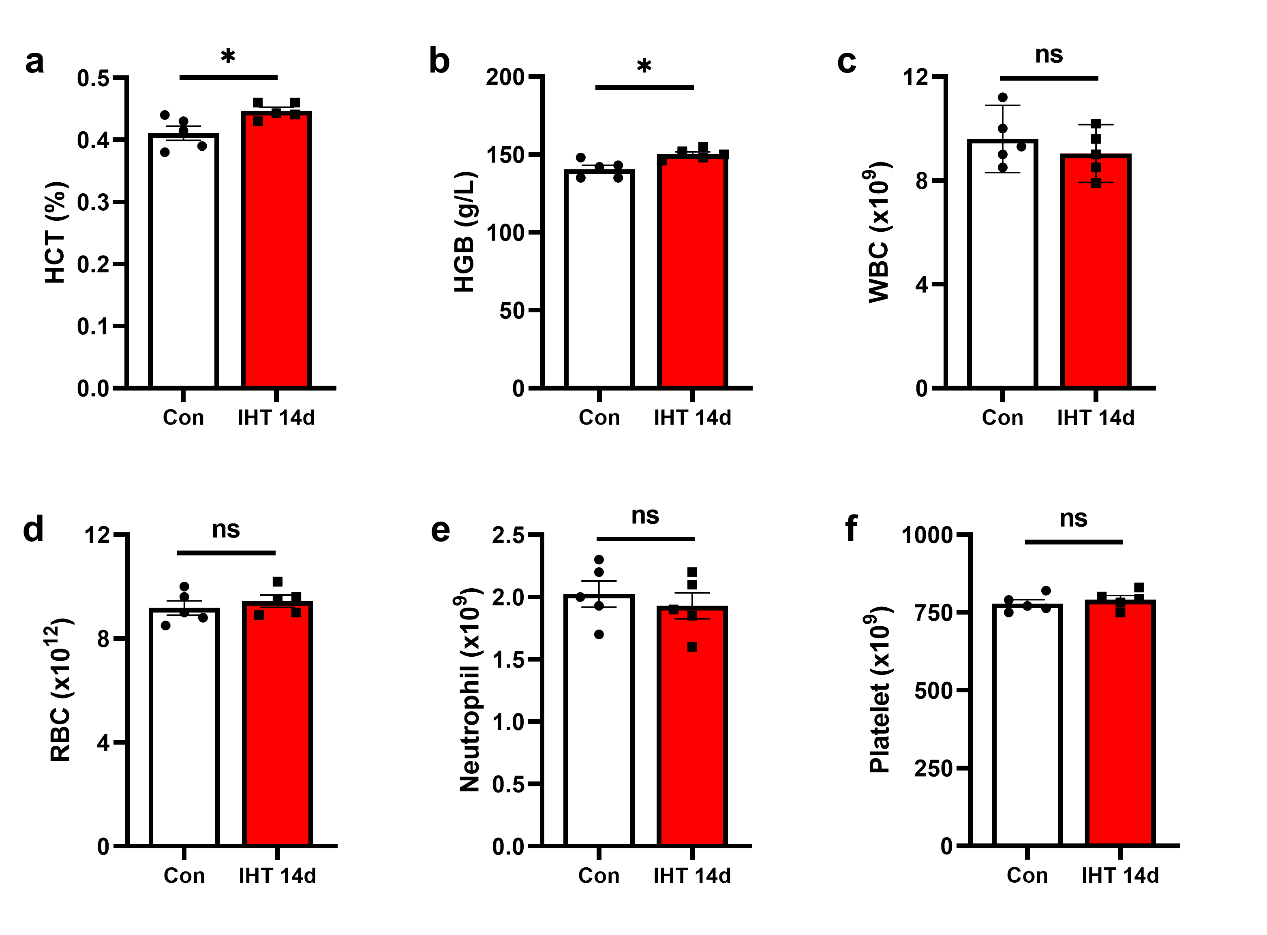


**Fig. S2** Effect of IHT on the blood test in APP/PS1 mice. There was no significant difference in (a) HCT: hematocrit, (b) HGB: hemoglobin, (c) WBC: white blood cell, (d) RBC: red blood cell, (e) Neutrophil, (f) Platelet in the blood of APP/PS1 mice with IHT 14d compared to the controls. n = 5 per group. ns, not significant.

**Figure. S3**


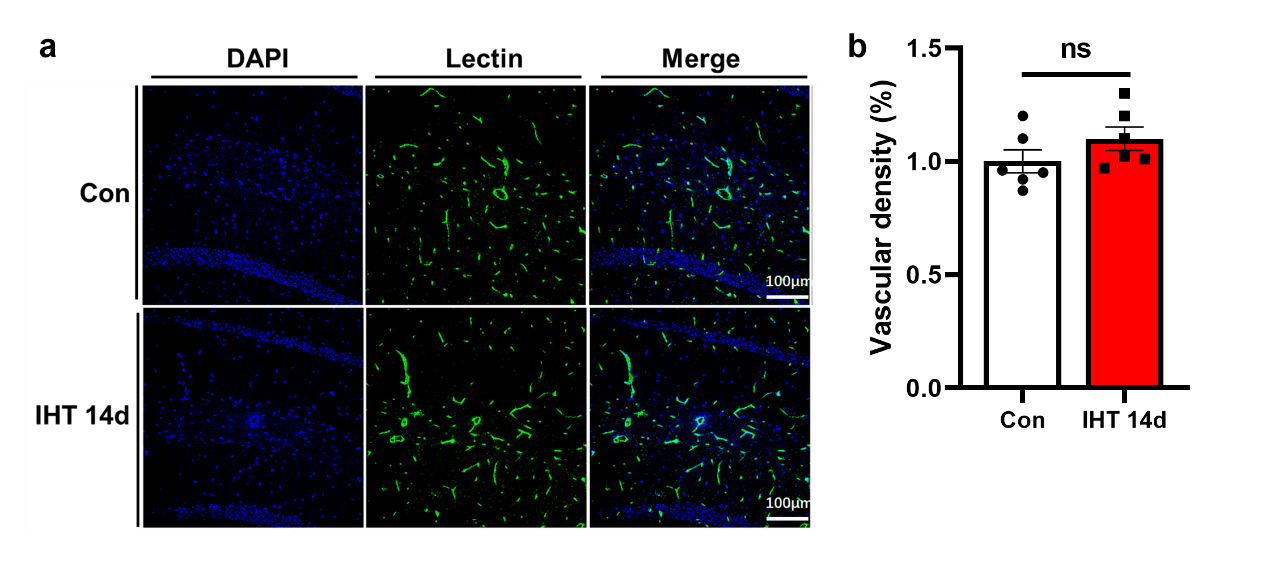


**Fig. S3** Representative immunohistochemical images stained with Lectin in APP/PS1 mouse brain sections. There was no significant difference in vascular density in the APP/PS1 mouse brain between the IHT 14d and the control groups. n = 6 per group. ns, not significant.

**Figure. S4**


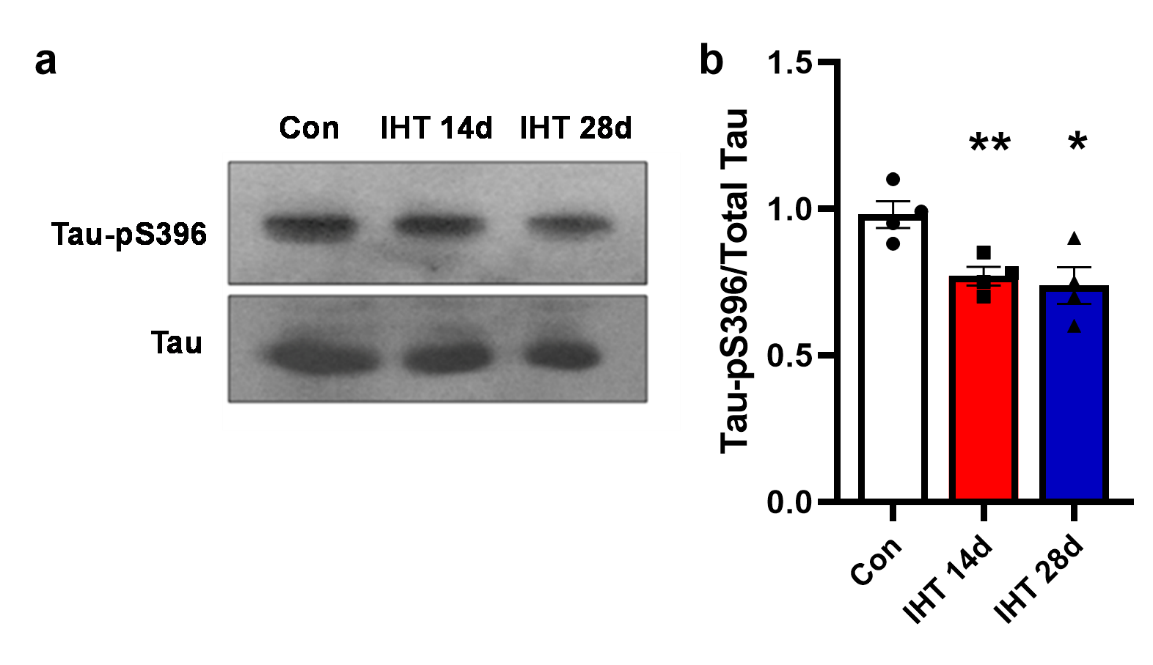


**Fig. S4** Representative Western blots of Tau-pS396 and total Tau in mouse hippocampus homogenate. There was significant decrease of the ratios of Tau-pS396 / total Tau protein expression level in APP/PS1 mouse brain between IHT 14d and 28d compared to the control groups. n = 4 per group; *p <0.05; **p < 0.01.

**Figure. S5**


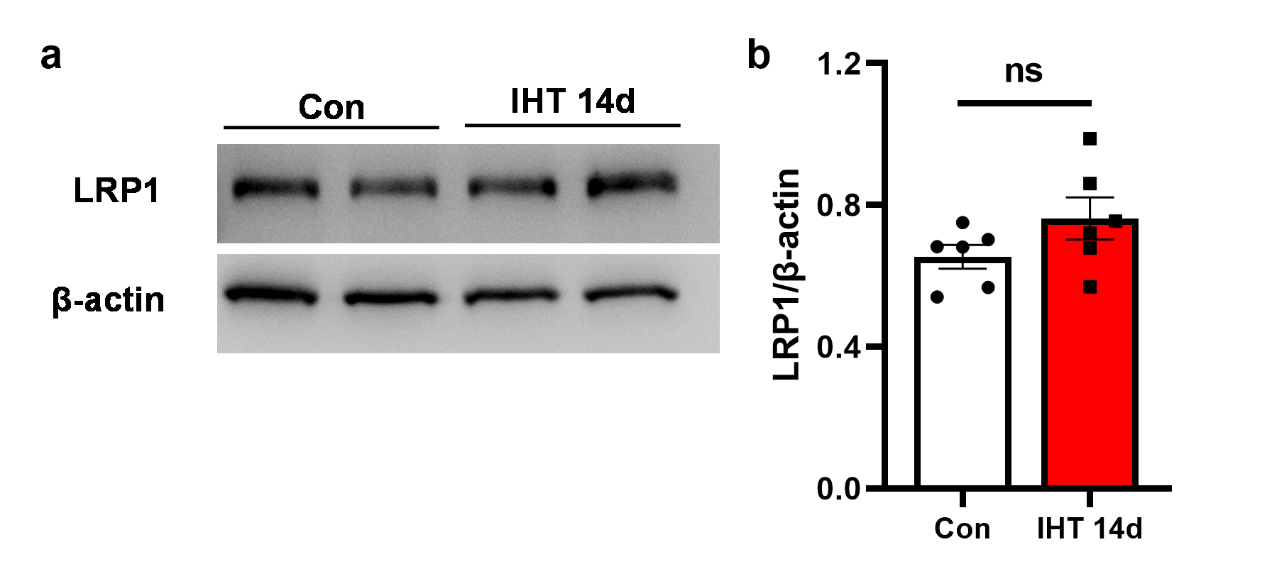


**Fig. S5** Representative Western blots of LRP1 in mouse hippocampus homogenate. There was no significant difference of the LRP1 protein expression level in APP/PS1 mouse brain between IHT 14d compared to the control groups. n = 6 per group. ns, not significant.
